# Recent Progress on Bio-Based Polyesters Derived from 2,5-Furandicarbonxylic Acid (FDCA)

**DOI:** 10.3390/polym14030625

**Published:** 2022-02-06

**Authors:** Xuan Fei, Jinggang Wang, Xiaoqin Zhang, Zhen Jia, Yanhua Jiang, Xiaoqing Liu

**Affiliations:** 1 Ningbo Institute of Materials Technology and Engineering, Chinese Academy of Sciences, 1219 Zhongguan West Road, Zhenhai District, Ningbo 315201, China; feixuan@nimte.ac.cn (X.F.); zxq@nimte.ac.cn (X.Z.); jiazhen8990@126.com (Z.J.); jyhua@nimte.ac.cn (Y.J.); 2 Key Laboratory of Bio-Based Polymeric Materials Technology and Application of Zhejiang Province, 1219 Zhongguan West Road, Zhenhai District, Ningbo 315201, China; 3 University of Chinese Academy of Sciences, No.19 A, Yuquan Road, Shijingshan District, Beijing 100049, China

**Keywords:** FDCA, homo-polyesters, co-polyesters, composites

## Abstract

The big challenge today is the upgrading of sustainable materials to replace miscellaneous ones from petroleum resources. Thus, a generic bio-based building block lays the foundation of the huge bio-market to green economy. 2,5-Furandicarboxylic acid (FDCA), a rigid diacid derived from lignocellulose or fructose, represents a great potential as a contender to terephthalic acid (TPA). Recently, studies on the synthesis, modification, and functionalization of bio-based polyesters based on FDCA have attracted widespread attention. To apply furanic polyesters on engineering plastics, packaging materials, electronics, etc., researchers have extended the properties of basic FDCA-based homo-polyesters by directional copolymerization and composite preparation. This review covers the synthesis and performance of polyesters and composites based on FDCA with emphasis bedded on the thermomechanical, crystallization, barrier properties, and biodegradability. Finally, a summary of what has been achieved and the issues waiting to be addressed of FDCA-based polyester materials are suggested.

## 1. Introduction

**Figure 1:**
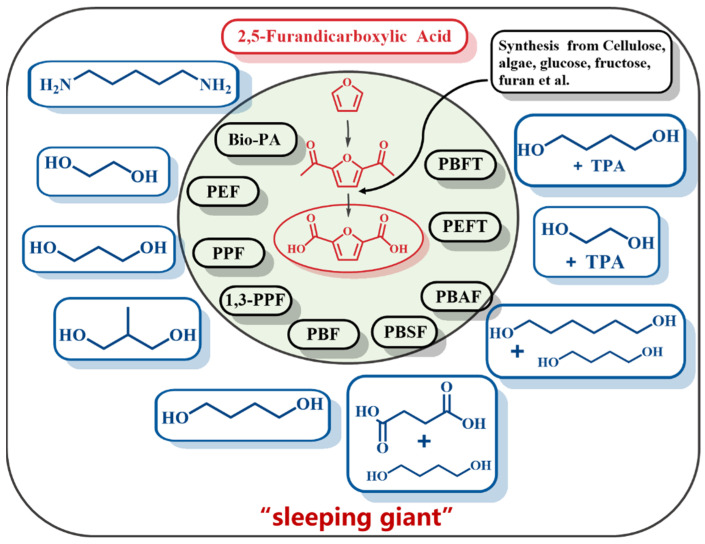
Different polyesters derived from 2,5-FDCA.

As concern regarding the pollution of petrochemicals spreads abroad, sustainability has been highlighted in the last decades: bio-based plastics from renewable resources have become a solution, trending within polymer research [1,2]. It is estimated that a total amount of 8000 Mt plastics has been produced worldwide, 79% of which has been discarded. Furthermore, the global production of plastics is rising stably, reaching 28,000 Mt in 2050 [3]. In this vein, the significance of recycling is highlighted as the European Parliament calls for a circular economy where waste prevention and recycling are encouraged. Currently, widely-applied bio-based polymers such as poly(lactic acid) (PLA) [4], polyhydroxyalkanoates (PHAs) [5], poly(glycolic acid) (PGA) [6], and polybutylene succinate (PBS) [7], which all belong to aliphatic polymers, lack rigidity from their innate structure, leading to weaker thermomechanical properties compared to oil-based poly(ethylene terephthalate) (PET), poly(butylene terephthalate) (PBT), polycarbonates (PC), etc. To move forward, bio-based polymer materials demand rigidity through polymerization or blending. Back in 2004, the U.S. Department of Energy presented “twelve platform bio-based chemicals”, among which FDCA was the only aromatic compound that was later employed heavily in the synthesis of polyester [8,9,10,11], polyamide [12,13,14,15], epoxy resin [16,17], and other polymers [18,19,20]. The large-scale investigations of polyesters have proven them to be promising bio-based building blocks that are expected to substitute TPA (Figure 1) [21,22,23]. Major corporations in the chemical industries such as DuPont and BASF have engaged in the mass production of FDCA [24]. In addition, the FDCA-based polyester of most interest, poly(ethylene 2,5-furancoate) (PEF), has been commercialized by Avantium with the YXY^®^ plant-to-plastics process [25].

FDCA has many reasons to explain its growing influence in the realm of bio-polymers. The primary one is the sustainable origin of FDCA. The main route is the oxidation of 5-hydroxymethylfurfural (HMF), which is produced from lignocellulose or fructose [26]. The fast development of renewable biomass undoubtedly promotes the downstream industry as Avantium, ADM, Shell, and BASF have engaged in the pilot plant production of HMF. One life cycle assessment showed that the non-renewable energy and the greenhouse gas emissions of PEF production are cut down by about 50% compared to that of PET [22]. Besides the green origin of FDCA, the foremost advantage is its structural similarity to TPA. The rigidity of its five-membered ring enhances the thermomechanical properties of polymers that can distinguish itself from other long-chain bio-monomers. Although the common features of rigidity from the ring structure are shared between FDCA and TPA, the divergences in the ring size, polarity, and geometry make differences in the macro performance of polyesters based on them. More details can be demonstrated as the interatomic distance between carboxylic acid groups in FDCA is 4.830 Å while in TPA, it is 5.731 Å [27,28]. The carbon atoms of the carboxyl units are non-linear in FDCA with an angle of 129.4° while TPA showed a linear structure. The structure, along with the polarity endowed by the oxygen atom, determines that the furan ring is more difficult to flip than TPA. These divergences produce a “butterfly effect” that greatly influences the macro-performances of furanic polyesters, resulting in the huge gap in thermomechanical, barrier, and anti-UV properties compared to TPA-based analogues.

Several reviews on polyesters based on renewable resources have been published, however, the discussion dedicated to furan-based polyesters with counterintuitive and interesting properties still seems helpful. In this paper, the synthesis and basic properties of homo-polyesters, co-polyesters, and composites based on FDCA were collected and discussed. The industrial construction was also involved. At the same time, the authors attempt to clarify the basal conditions and probe the next step of the investigation into furan-polyesters.

## 2. Synthetic Routes of Furanic Polyesters

**Scheme 1:**
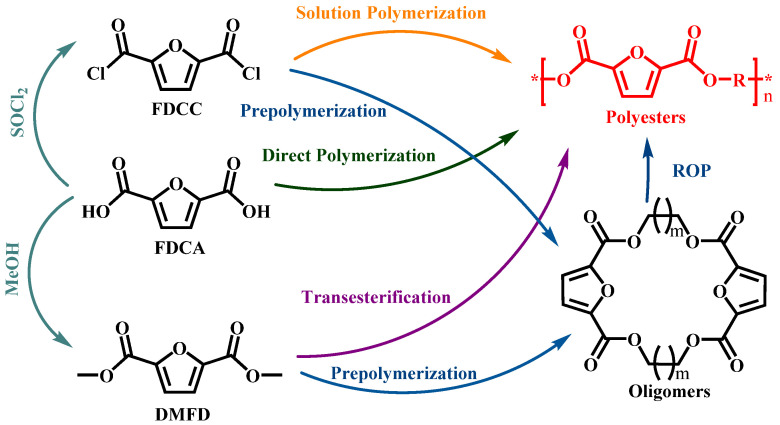
Synthesis of FDCA-based polyesters from melt polymerization, solution polymerization, and ROP.

**Figure 2:**
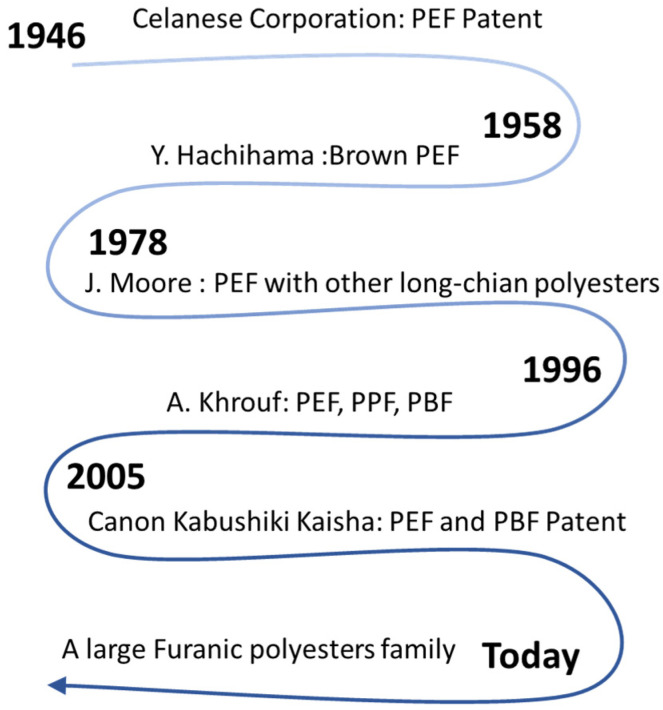
The history of PEF and synthesis of other furanic polyesters.

Three ways are widely accepted as polyester synthetic routes: melt polymerization, solution esterification, and ring-opening polymerization (ROP) (Scheme 1). Melt polymerization can be further split across two methods based on the starting materials: FDCA with direct polymerization or dimethyl furan-2,5-dicarboxylate (DMFD) from transesterification. Industrial production favors direct esterification to avoid an additional esterification step while transesterification is preferred in lab synthesis to produce polyesters with novel structures. In the early exploration of furanic polyester synthesis, solution polymerization was tried, but suffered from multiple steps of isolation and purification, low yield, and low molecular weight, etc. However, considering that sometimes the melting point of the product is too high, solution polymerization is an option. ROP have been reported more recently to obtain polyesters from cyclic esters or anhydrides [29,30]. Additionally, ROP is the mainstream in the industrial production of PLA. The first report of furanic polyester synthesis can be dated back to 1946 when the Celanese Corporation of America requested a patent application for FDCA transesterification synthesis of PEF [31]. Next, with many explorations and dedication into FDCA-based polyester synthesis, the investigations gathered momentum by around 2010 (Figure 2).

## 3. Homo-Polyesters from FDCA

### 3.1. PEF

**Figure 3:**
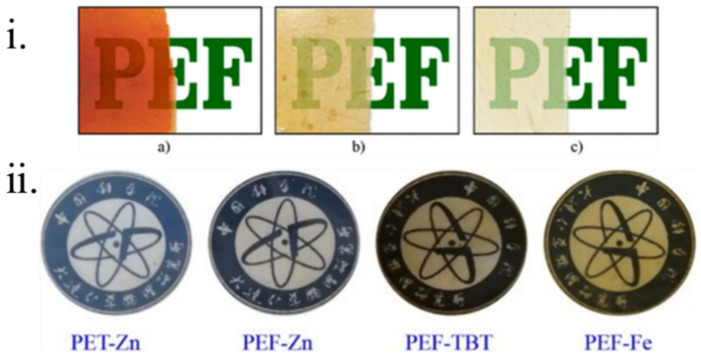
(**i**) 70 μm films of PEF (**a**) TBT 400 ppm; (**b**) ZnAcO 400 ppm; (**c**) Al(acac) 350 ppm [35]; (**ii**) Appearance comparison of PET catalyzed by metal Zn and PEFs catalyzed by metal Zn, TBT, and Fe [36].

As a top candidate in the race between bio-based and petroleum-based polyesters, PEF is definitely worth specific discussion. In 2009, Gandini et al. [9] reported a pioneer work where a variety of methods were involved in PEF synthesis. Following this, more reports of PEF properties demonstrated the potentiality and possibility of PEF: its good thermomechanical behaviors and excellent barrier property. In 2011, Gomes et al. [10] prepared PEF via transesterification. The glass transition temperature (T_g_) was measured as 80 °C, higher than that of PET (75 °C). The melting temperature (T_m_) was given as 215 °C, much lower than that of PET (260 °C). The authors suggested that PEF may replace PET based on the satisfied thermal performances. Zhou’s group [11] investigated the mechanical properties of PEF and found a tensile modulus of 2100 MPa and tensile strength of 66.7 MPa, which was close to that of PET (2000 MPa and 45 MPa). These early investigations of the thermomechanical properties of PEF greatly inspired more in-depth studies. PEF synthesized by ROP was proved feasible, and a weight–average molecular weight (M_w_) was reached of 50,000 g/mol. The thermal properties were similar to those obtained via melt polymerization [32]. Knoop et al. [33] prepared PEF via transesterification and solid-state post-condensation (SSPC) to obtain high molecular weight PEF (number-average molecular weight: 83,000 g/mol). The tensile modulus and strength were determined as 2450 MPa and 35 MPa, respectively. In the SSPC process, the solid polyester phase was dispersed in a gaseous phase or liquid phase to continue the transesterification to avoid the unwanted thermal oxidative degradation caused by the long reaction time of traditional melt condensation. The temperature and reaction time were the crucial factors in SSPC: on one hand, the kinetics of chain end diffusion would be slowed down so the long time is required to obtain the designed molecular weight, which would be inefficient for industrial production. On the other hand, the high temperature demands the high crystallinity of polyesters to stay solid, which further shrinks the amorphous proportion, thus limiting the mobility of the reacting groups. In a word, the SSPC process exhibits high complexity so that the conclusions of related reports is sometimes controversial [34]. Banella et al. pursued targeted synthesis of PEF suitable for food-contact-grade packaging material by applying zinc acetate and aluminum acetylacetonate as catalysts. The results showed that both catalysts were operative in the PEF preparation, resulting in film production with high transparency and low discoloration (Figure 3i) [35]. Zhou et al. [36] synthesized white PEF with a fast crystallization rate using metal zinc as the catalyst via transesterification (Figure 3ii). They found that the new catalyst inhibited the discoloration and enhanced the crystallization rate. Besides the basic thermal and mechanical properties, Koros et al. [37,38] explored the barrier properties of PEF and outlined a variety of important conclusions: compared to PET, PEF had an 11 and 19 times higher O_2_ and CO_2_ barrier and the reason behind this was ascribed to the difficulty of furan-ring flipping due to the furan ring polarity and the structural nonlinearity [39]. Liu et al. [40] tried to explain the surprisingly good barrier properties of furan-based polyesters from the role of the furan ring’s polarity. Results showed that although PEF had a higher free volume than poly(ethylene-2,5-thiophenedicarboxylate), it still exhibited a better barrier property, which was attributed to the higher dipole moment of the furan ring. The basic thermal and mechanical properties of PEF are collected in Table 1.

The industrial production of PEF is guided by Avantium, who built a pilot plant in the Netherlands to produce PEF via its famous YXY^®^ plant-to-plastics technology. They plan to open its flagship factory in 2023 with the expected capacity of five kilotons per year [43]. The PEF commercialization in Asia is under the plan of Mitsui (Japan) who had a partnership with Avantium. Recent news of ETH Zürich offering another option in the large-scale production of PEF besides melt polymerization: they reported the rapid synthesis of bottle-grade PEF from cyclic oligomers. Sulzer is working with them to adjust the process to adapt for an industrial application [44,45].

### 3.2. Furan-Polyesters with Aliphatic Diols

Aliphatic long-chain diols (C2–C18) employed in the synthesis of other poly(alkylene furanoates) have been widely reported [10,46,47,48]. Zhou et al. [11] successfully synthesized a series of homo-polyesters from C3 to C8 via direct polymerization, namely, poly(1,3-propylene 2,5-furandicarboxylate) (PPF), poly(1,4-butylene 2,5-furandicarboxylate) (PBF), poly(1,6-hexylene 2,5-furandicarboxylate) (PHF), and poly(octylene 2,5-furandicarboxylate) (POF). The T_g_ and T_m_ were measured as 21.8–89.9 °C and 148.2–210.4 °C while the tensile modulus and elongation at break ranged from 340 to 2070 MPa and from 4.2 to 210%. Later, Bikiaris et al. [42] further synthesized polyesters with longer diols such as C8, C9, C10, and C12. The characterization of these more ductile polyesters showed a T_g_ under 0 °C and inferior mechanical properties. When the length of the aliphatic chain increased, the T_g_ of these polyesters continuously declined, as listed in Table 1. Besides the synthesis methods above-mentioned, PAF preparation via enzyme polymerization was also reported [49]. However, most number-average molecular weights (M_n_) of these polyesters were low (200–10,100 g/mol) and characterization of properties was absent.

**Figure 4:**
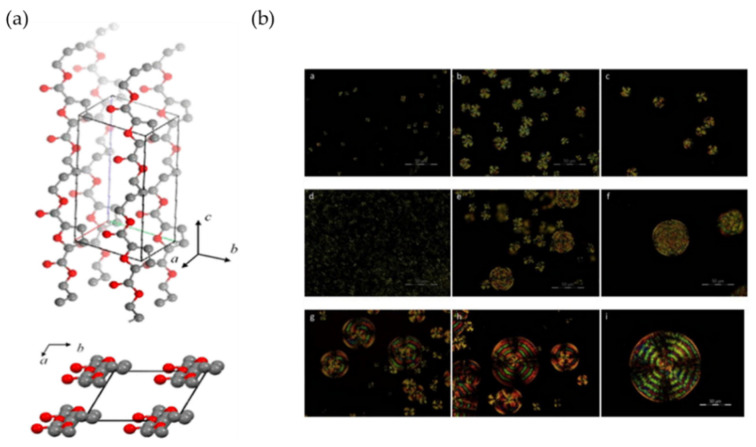
(**a**) Views of the PBF structure [21]; (**b**) PLM images of spherulites of (a) PPF grown at 130 °C, (b) PPF grown at 135 °C, (c) PPF grown at 145 °C, (d) PPN grown at 165 °C, (e) PPN grown at 175 °C, (f) PPN grown at 190 °C, (g) PPT grown at 208 °C, (h) PPT grown at 212 °C, (i) PPT grown at 214 °C with a scale bar of 50 μm [41].

In fact, PBT and PPT are famous engineering plastics, so studies on their furan-based homologs, PBF, and PPF have been broadly reported. Zhu et al. [21] investigated the thermal and mechanical properties of PBF and compared them to that of PBT, concluding that the good thermal stability and mechanical strength of PBF made it a possible alternative to PBT. When the M_w_ of PBF reached 65,000 g/mol, the T_g_ and T_m_ were 40 °C and 173 °C, respectively. Meanwhile, the tensile modulus was around 1000 MPa. Via wide angle X-ray scattering, the structure of PBF (Figure 4a) was found to be close to the α and β forms of PBT. Ma et al. [50] also contended that PBF may be a furan counterpart of PBT after investigating their crystallinity and found that the PBF filament could be drawn directly from the melt. Thiyagarajan et al. [51] investigated the thermal properties of PBF from 2,5-FDCA, 2,4-FDCA, and 3,4-FDCA. Results showed that no significant influence was found in T_g_. The synthesis, thermal, and mechanical properties of PPF have also been reported [10,11], which showed that PPF has sufficient mechanical strength and thermal stability such as 1500 MPa for tensile modulus and 68 MPa for tensile strength [11] as well as the degradation temperature of 390 °C [10]. Papageorgiou et al. [41] made a comparison between PPF, poly(propylene terephthalate) (PPT), and poly(propylene 2,6-naphthalate) (PPN) in a thermal analysis and found that the thermal transition temperatures of PPF were similar to that of PPT. Additionally, the polarized light microscopy signaled that the size of the PPF crystal was smaller (Figure 4b) and the crystallization of PPF was slower than that of PPT. Zhao et al. [52] compared the barrier properties among PEF, PPF, and PBF theoretically and experimentally. They found that for PEF and PPF, there was little difference in barrier property, however, as the aliphatic chain increased (PBF), the oxygen permeability showed a significant increase while the CO_2_ permeability remained unchanged. The collection of the thermal and mechanical properties of these polyesters are listed in Table 1.

### 3.3. Furan-Polyesters with Rigid Diols

**Scheme 2:**
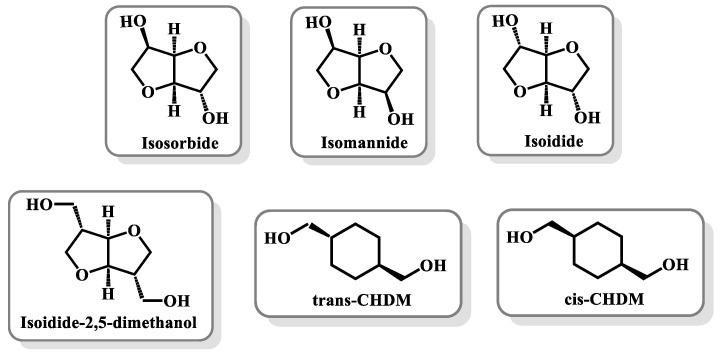
Structure of rigid diols in polyester synthesis.

One of the important issues of aliphatic long-chain polyesters is the low T_g_, which dramatically reduces its application temperature range. To impart the desired stiffness to furan-polyesters, 1,4:3,6-dianhydrohexitols (DAHs), which have three stereoisomers according to the stereochemistry of the two hydroxyl groups, have been employed in many reports [53,54,55,56]. Among them, isosorbide (ISB) is the most widely studied and has been employed in commercial biopolymers as the obviously rigid structure and green origin. The polyester from FDCA and isosorbide (PDASF) or isoidide (PDAIF) were prepared via solution polycondensation. Their T_g_ reached 180 °C and 140 °C, respectively [10]. Lopez-Sanchez et al. [57] synthesized fully biomass-derived polyester coatings from FDCA, ISB, succinic acid, 1,3-BDO, and 1,5-PDO. ISB was introduced to increase T_g_ by approximately 40 °C. Terzopoulou et al. [58] synthesized poly(isosorbide furanoate) (PIsF) from DMFD and ISB via melt polycondensation for a food packaging application. Results showed that the T_g_ was 157 °C and T_dmax_ was 421 °C with an intrinsic viscosity of 0.39 dL/g. Chebbi et al. [59] synthesized poly(decamethylene-co-isosorbide 2,5-furandicarboxylate)s (PDIsFs) from DMFD, ISB, and 1,10-decanediol via melt polycondensation with M_n_ within the range of 11,500–25,400 g/mol. ISB units can obviously enhance the thermal performance (T_g_ of −1 °C to 21 °C, T_d5%_ of 405 °C to 413 °C) and mechanical properties (tensile modulus ranging from 14 MPa to 559 MPa and elongation at break between 205–266%) of the obtained polyesters. Chen et al. [60] employed ISB to modify PBF. The T_g_ of copolyesters reached 107 °C with ISB moiety of 50%. In addition, the hydrolytic degradation was promoted by ISB content. Wang et al. [61] synthesized fully bio-based poly(isosorbide-co-butylene 2,5-furandicarboxylate) (PISBF) from DMFD, ISB, and 1,3-BDO. The T_g_ and T_dmax_ of PISBF was between 55 °C and 150 °C and 405 °C and 417 °C, respectively. Results showed that BDO toughened the poly(isosorbide 2,5-furandicarboxylate) (PIF) polyester chains where the elongation at break was higher than 46%. Kasmi et al. [62] synthesized poly(1,4-cyclohexanedimethanol-co-isosorbide 2,5-furandicarboxylate)s (PCIsFs) from DMFD, ISB, and CHDM with T_g_ ranging from 76.8 °C to 103.5 °C. The investigation of crystallization revealed that isosorbide furanoate were inserted in the crystals of PCF homo-polyester. Besides FDCA-based polyesters, ISB has been introduced in numerous polymers. Wu et al. [63] employed isoidide dicarboxylic acid (IIDCA) and diols derived from isohexide building blocks to synthesize novel bio-polyesters. The obtained polyester from IIDCA and ISB (PIsI) showed a T_g_ of 73 °C and T_d5%_ of 274 °C with M_n_ of 2600 g/mol while polyester (PImI) from IIDCA and isomannide (IM) showed a T_g_ of 30 °C and T_d5%_ of 267 °C with M_n_ of 1200 g/mol. For polyester (PIiI) from IIDMC and isoidide (II), the observed T_g_ and T_d5%_ were highest at 80 °C and 286 °C with M_n_ of 2500 g/mol. The authors suggested that IIDCA can enhance the T_g_ by approximately 70 °C, as introduced into the polymer main chain. Jacquel et al. [64] modified PBS polymers with ISB (PBIS) or FDCA (PBSF) for biodegradable film applications. The thermal properties of the copolyesters obtained were compared to PBST. TPA, FDCA, and ISB all showed positive effects on Tg, among which ISB exhibited a 19 °C increase in T_g_ compared to pristine PBS (−30 °C). Furthermore, PBSFs were observed as compostable polyesters. Wu et al. [65] prepared poly(isoidide-2,5-bismethylene furan-2,5-dicarboxylate) (PXIIF) from extended isoidide, isoidide-2,5-dimethanol, via melt polycondensation. The M_n_ of the obtained polyesters was 6900 g/mol and later reached 30,300 g/mol after SSPC. The T_g_ from the DSC results was 94 °C and the T_m_ was 250 °C. Other cyclic compounds such as 1,4-cyclohexanedimethanol (CHDM) and 1,4-cyclohexanedicarboxylic acid (CHDA) were also preferred in polyester preparation [23,66,67,68,69]. Matos et al. [66] undertook a comparative study between poly(1,4-cyclohexylene 2,5-furandicarboxylate) (PCdF) and poly(1,4-cyclohexanedimethylene furandicarboxylate) (PCF). The high T_g_ of PCdF (175 °C) was attributed to the increasing stiffness of the linkage between cyclohexylene and the furan ring. Wang et al. [23] investigated the different steric conformation of PCF and found that the thermal and mechanical properties were heavily affected. When the *trans* moiety of CHDM increased, the T_m_ and T_g_ increased from 219 °C to 291 °C and 71 °C to 87 °C, respectively. Additionally, PCF-*trans* 98 exhibited the shortest crystallization half time at 7 s and the best mechanical properties (1820 MPa for tensile modulus and 52 MPa for tensile strength). Other cyclic monomers were also investigated and patented such as 2,2,4,4-tetramethylcyclobutane-1,3-diol [70], dianhydrohexitols [71], hydroquinone [72], and dichloro-2,3-o-isopropylidene L-tartrate [73]. Scheme 2 shows the structure of the main rigid diols above-mentioned. As shown in Table 2, the thermal and mechanical properties of polyesters with rigid diols are listed. It is worth mentioning that the mechanical information was absent for most of the references due to the brittleness of the main chain.

## 4. Co-Polyesters from FDCA

### 4.1. Balance between Rigidity and Flexibility

Taking PEF as an example again, as much interest has been garnered due to its promising properties compared to its terephthalate homolog. However, the brittleness and low crystallization rate limited the further application of PEF. Its modification is to soften the fragile chain and enhance the crystallizability, thus increasing the crystallization rate and the elongation at break [33,74]. As discussed previously, furan-polyesters with long-chain aliphatic diols exhibited improved elongation at break and crystallizability, nevertheless, their thermal transition temperature and mechanical properties deteriorated. It can be assumed that if PEF was softened by the incorporation of the linear diols, the originally good T_g_, T_m_, mechanical strength, and modulus would decline to substandard. Ma et al. [75] combined two frequently-used diols, ethylene glycol (EG) and 1,4-butylene glycol (BG), to prepare the poly[(ethylene 2,5-furandicarboxylate)-co-(butylene 2,5-furandicarboxylate)] (PEF-PBF). The thermal characteristics showed that the T_g_ decreased as the BG content increased. They confirmed that diols with longer chains exhibited higher reactivity with FDCA while the mechanical property was absent.

The cyclic monomers were found to be well balanced between the required high T_g_ and the need to toughen the PEF chain. In the report by Hong et al. [76], CHDM was employed in the synthesis of PEF via transesterification. Results showed that the elongation at break was increased to 79% as CHDM content increased, thus confirming that CHDM improved the ductility of the backbone. Naturally, the crystallization rate was also enhanced upon the introduction of CHDM as the additional strain-induced crystallization was observed in the tensile test. Then, after SSPC section, the polyesters obtained were successfully processed as bottles, which exhibited better oxygen permeability and less acetaldehyde content compared to PET-containing bottles. Liu et al. [77] prepared poly(ethylene-co-1,4-cyclohexanedimethylene 2,5-furandicarboxylate)s (PECFs) via transesterification and the products exhibited enhanced toughness with satisfying thermal and mechanical properties. The crystallizability and mechanical properties increased with the increasing CHDM moiety. Poly(ethylene 2,5-furandicarboxylate-co-ethylene 1,4-cyclohexanedicarboxylate)s (PEFCs) were also synthesized and investigated [78]. The thermal characteristics showed that the T_g_ decreased with the increasing content of CHDA and all samples showed thermal stability at 350 °C. Results showed that PEFC-30 behaved as a toughened polyester, since tensile modulus was 800 MPa and the elongation at break was more than 600%. Poly(butylene-co-1,4-cyclohexanedimethylene-2,5-furandicarboxylic acid) (PBCFs) were prepared via direct esterification. Thermal characteristics showed that the T_g_ (45.7–87.5 °C) and T_m_ (140.1–264.9 °C) of the polyesters obtained increased with the increasing CHDM moiety. The crystallinity and crystal structure were also affected by CHDM content as when the CHDM moiety increased, the polyesters became semi-crystalline from completely amorphous [79]. Jia et al. [80] employed CHDM to modify PPF. The co-polyesters showed satisfying good mechanical properties, although the barrier performance weakened a little. Terpolyesters based on FDCA, ISB, EG, and CHDM (PEICF) were prepared via direct esterification [81]. The polyesters obtained were all completely amorphous since no T_m_ was observed. The T_g_ increased linearly as the increasing isosorbide (ISB) moiety and the maximum one reached 182.4 °C when the ISB content was 100%. Poly(propylene-co-1,4-cyclohexanedimethylene 2,5-furandicarboxylate)s (PPCFs) showed enhanced T_g_ (59–73 °C) than PPF (56 °C) and comparable mechanical properties as elongation at break was over 150% for all co-polyesters [80]. 2,2,4,4-Tetramethyl-1,3-cyclobutanediol (CBDO) was another unique cyclic diol that has been employed in PEF modification [82]. The poly(ethylene-co-2,2,4,4-tetramethyl-1,3-cyclobutanediol 2,5-furandicarboxylate) (PETF) showed satisfying properties: T_g_ was around 90 °C and tensile modulus reached 3000 MPa with elongation at break of 10%. Additionally, the co-polyesters films showed high transparency with seven times and five times better barrier property for CO_2_ and O_2_ compared to PET. Zhou et al. [83] prepared poly(ethylene 2,5-furandicarboxylate)-poly(ethylene glycol) (PEFEGs) based on PEF and poly(ethylene glycol) (PEG). The T_g_ of the polyesters obtained was from 78 °C to 85 °C with an elongation at break up to 60%. With the increasing content of PEG, the shape fixity increased. They also tried to synthesize a series of furfural-based quadri-copolyesters based on FDCA, BDO, succinic anhydride (SA), and tricyclic diacid (TCDA) as an alternative to PBT. The polyesters obtained showed a satisfied T_g_ (36.7 °C) and tensile strength (46.9 MPa) with excellent ductility (elongation at break of 830%) [84]. Gao et al. [85] employed alicyclic (1R,3S)-1,2,2-trimethylcyclopentane-1,3-dimethanol (TCDM), which was derived from camphor in the polyester synthesis with linear or aromatic diacids. Polyesters based on FDCA showed a high T_g_ of 98 °C. Liu et al. [86] synthesized a series of novel polyesters with T_g_ higher than 100 °C from FDCA, BDO, and bis [4-(2-hydroxyethoxy) phenyl] sulfone (BHEPS). When the BHEPS content reached 65%, the T_g_ was 104.7 °C with M_w_ of 28,500 g/mol, tensile strength of 82 MPa, and elongation at break of 98%. Table 3 summarizes the properties of these polyesters, as discussed above.

### 4.2. Introduction of Biodegradability

Most FDCA homo-polyesters lack biodegradability. The introduction of monomers such as ε-caprolactone (CL), adipic acid (AA), and succinate to furan-polyesters, is a simple way to impart biodegradability to furanic polyesters. The co-polyesters based on these monomers and TPA have shown good biodegradability as poly(butylene adipate-coterephthalate) (PBAT) [87,88] and poly(butylene succinate-co-terephthalate) (PBST) [89,90].

CL is a famous building block of semi-crystalline polyester poly(ε-caprolactone) (PCL), which has been widely applied in the biomedical field due to its superior biodegradability, biocompatibility, and drug permeability [91,92,93]. Poly(ethylene 2,5-furandicarboxylate-co-ε-caprolactone) (PEFCL) was synthesized and the thermomechanical properties were discussed [94]. The T_g_ was found to decrease with the increase in CL moiety. The mechanical modulus and strength decreased slightly while elongation at break enhanced notably when the CL content continued growing. However, information on biodegradability was absent. Kasmi et al. [95] investigated CL-based furan-polyesters from 1,5-pentanediol and 1,6-hexanediol via ROP. The poly(pentylene 2,5-furandicarboxylate-co-caprolactone) (PPeCFs) and poly(hexamethylene 2,5-furandicarboxylate-co-caprolactone) (PHeCFs) were thermally stable at 310 and 360 °C, respectively. PPeF and PHeF degraded very slowly, which remained invariant after 25 days of testing, while the incorporation of over 40 mol% CL content obviously accelerated degradation as 32% and 13% weight loss was achieved by PHeCF 50/50 and PHeCF 60/40, respectively. Zheng et al. [96] synthesized an elastomer, poly(butylene 2,5-furandicarboxylate-ε-caprolactone) (PBFCL) via ROP with randomly distributed rigid and soft segments. The polyesters obtained showed good mechanical strength (25 MPa) and thermal stability (T_d,5%_ > 320 °C). The CL content ensured the biodegradability of polyesters, however, the crystalline part hindered the enzymatic attack. The results confirmed that by simply introducing the CL units to the polyester, biodegradability could be obtained. Morales-Huerta et al. [97] prepared poly(ε-caprolactone-co-butylene 2,5-furandicarboxylate) by enzymatic ROP with Lipase Candida Antarctica (CALB) as the catalyst. The advantages of enzymatic ROP can be concluded as mild conditions, no volatile by-products, and low coloration with the M_w_ between 22,000–50,000 g/mol. The BF content increased the thermal transition temperature of co-polyesters while slowing down the crystallization rate. The result clarified that the co-polyesters obtained remained unchangeable in the hydrolytic degradation while exhibited notable weight loss (ranging from 20% to 40%) in the presence of lipases. Hu et al. [98] prepared poly(butylene furandicarboxylate-co-ε-caprolactone) (PBFCL) co-polyesters where the results showed that after 48 days with enzymes, the weight loss could be over 30% and when the CL content was more than 40 mol%, the co-polyesters exhibited good recoverability.

Succinate and adipate are also common monomers for co-polymerization of furan-polyesters. The aliphatic-aromatic co-polyesters employing succinate such as PBST and poly(butylene succinate-co-2,5-furandicarboxylate) (PBSF) exhibited the desired biodegradability and good thermomechanical properties [99,100,101]. Poly(butylene 2,5-furandicarboxylate-co-succinate) (coPBF_x_S_y_) was synthesized via ROP [102]. Enzymatic degradation revealed that the succinate moiety could improve the degradation process while PBF remained stable under the same conditions. Poly(1,3-propylene 2,5-furandicarboxylate-co-1,3-propylene succinate) (PPFPS) synthesized via polycondensation and azeotropic distillation have been investigated and a kinetic model for furan-polyesters was suggested [103]. Results showed that the rate of polymerization would be promoted by the elevation of temperature as expected, while at around 230 °C, the polymerization rate was decreased due to the increasing monomer evaporation. Wu et al. [104] also employed succinate in the polymerization of PBF. Results showed that PBSF was less crystallizable than poly(butylene succinate), but still had fast crystallization behavior in isothermal and non-isothermal characterization. The elongation at break, tensile modulus, and tensile strength were 230–758%, 330–680 MPa, and 18–56 MPa, respectively. The information of degradation was not discussed. Later, the same group [105,106] synthesized and discussed the degradation behavior of poly(butylene adipate-co-butylene furandicarboxylate) (PBAF) and poly(butylene succinate-co-butylene furandicarboxylate) (PBSF). Results showed that in the 110 day composting test, all samples accomplished more than 90% biodegradability. Unsurprisingly, higher BF content led to slower degradation as the alkaline conditions would accelerate the weight loss more than acidic and neutral conditions due to the better water solubility and diffusibility of degradation products. Compared to their TPA-based homologs, the furan-polyesters were easier to degrade. Hu et al. [107] synthesized a series of poly(propylene succinate-co-furandicarboxylate) (PPSF) ranging from poly(propylene succinate) (PPS) to PPF. It is clear that degradation would be improved with the increase in succinate content as PPSF60 exhibited the lowest weight loss of 3.5% and the weight loss of PPSF10 was 14.8%. Besides, the co-polyesters obtained showed good mechanical performance and excellent barrier property, which determined its potential for green packaging applications. Adipate has been successfully introduced in the synthesis of TPA-based plastics that reached the perfect combination of mechanical property and biodegradability such as poly(butylene adipate-co-butylene terephthalate), which has been commercialized as Ecoflex^@^ and Easter-bio^@^ [108,109]. Therefore, it is of interest and promise to introduce adipate into FDCA-based polyesters targeting biodegradability and tough products. Papadopoulos et al. [110] prepared co-polyesters based on PEF and poly(ethylene adipate) (PEAd) with good thermal stability as T_d_ was over 300 °C. Results showed that when the content of ethylene adipate was over 90%, the co-polyesters could degrade completely after a month. Zhou et al. [111] investigated poly(butylene adipate-co-butylene furandicarboxylate (PBAFs), which showed biodegradability with FDCA content at a range of 0–50 mol%. When the FDCA moiety rose to 75%, the co-polyesters were un-degradable. Wu et al. [112] conducted a comparative study between PBF and PBAF. Results showed that PBAFs were all semi-crystalline polyesters and manifested high-elastic deformation with BF content under 50 mol%. With higher BF moiety, PBSFs performed as non-rigid plastics (42–110 MPa and 30–42 MPa for tensile modulus and strength) while PBF behaved as tough plastics (1900 MPa and 56 MPa for tensile modulus and strength). Both showed high elongation at break (>250%).

Other biodegradable monomers have also been investigated in the polycondensation of furan-polyesters. Hu et al. [113] synthesized poly(butylene furandicarboxylate-co-glycolate) (PBFGA) via melt polycondensation. The polyesters obtained showed good thermal stability (>320 °C), mechanical properties (500–1260 MPa for tensile modulus and 16–44 MPa for tensile strength), and over 220% for elongation at break while exhibiting a superior gas barrier property to the commercial PBAT films. Furthermore, the enzymatic degradation test showed an obvious weight loss when glycolic acid content was more than 20 mol%. Soccio et al. [114] synthesized poly(butylene 2,5-furanoate/diglycolate) P(BF_x_DG_y_) random co-polyesters via melt polycondensation. Results showed that the samples exhibited higher thermal stability (T_max_ > 380 °C) than PBF (T_max_ = 366 °C). The degradation rate of the co-polyesters depended strictly on the composition and crystallinity, where P(BF_60_DG_40_) showed more than 40% weight loss after 62 days while P(BF_90_DG_10_) showed about 10% weight loss under the same conditions. The mechanical properties deteriorated with the increasing DG units. Wu et al. [115] prepared co-polyesters based on lactic acid and ethylene glycol (PEFL). The T_d_ ranged from 298 °C to 398 °C since more EF content would enhance the thermal stability. After 55 days in PBS buffer solution and in soil, the PEFL20/80 exhibited weight loss of 24% and 65%, respectively. Jia et al. [116] completely synthesized amorphous co-polyesters from EG and dodecanedioic acid (DDCA) via transesterification. The elongation at break was over 200% for all samples except for PEDF-10. PEDF-60 exhibited the best biodegradability of over 5% weight loss while PEF and PEDF-20 showed no weight loss during the 28 day test. Polyester based on 2,5-furandicarboxylic acid and a very-long aliphatic monomer, 1,20-eicosanediol, was synthesized and investigated [117]. The incubation result showed that after 21 days, the poly(1,20-eicosanediyl 2,5-furandicarboxylate) lost about 11% weight in the presence of *Porcine pancreas* enzyme. Weinberger et al. [118] investigated the PEF enzymatic degradation and found that the influence of particle size became smaller when the molecular weight was higher and they optimized the hydrolysis conditions such as in the 1 M phosphate buffer, pH 8, at 65 °C with *Humicola insolens*, where PEF reached 100% hydrolysis. Zhou et al. [119] synthesized a series of biodegradable polyesters with FDCA, BDO, and oxabicyclic units via ROP. The polyesters obtained showed biodegradability within seven days with less than 30 mol% FDCA units. Furthermore, the polyesters exhibited good mechanical (34 MPa of tensile strength) and barrier properties. Koo et al. [120] synthesized poly(butylene adipate-co-furanoate) (PBAF) and compared the properties with PBAT. PBAF showed instant elastic recovery in the stretching test while PBAT malfunctioned. In the enzymatic degradation, only the PBAF with 50% FDCA went through hydrolysis due to the specificity of the enzyme.

## 5. Composites of Furan-Polyesters

### 5.1. Inorganic and Organic Fillers

Polymer composites promoted by the advances of nanotechnology have been regarded as an indispensable part of polymer sciences. In fact, nanocomposites have been widely applied in the biomedical, electronic, automotive fields, etc. [121,122]. As furan-polyesters with such great potential have manifested, the field has been further extended by the evaluation of furan-contained composites. In fact, carbon-based composites of polymers have been reported to enhance the thermal, mechanical, and electrical properties [123,124,125,126]. Multi-walled carbon nanotubes (MWCNTs) and graphene oxide (GO) have been employed in the preparation of PEF nanocomposites, which showed faster crystallization than the neat material due to the higher nucleation density [127]. Generally speaking, a good dispersion of nano-fillers results in elevated nucleation sites, thus explaining the increasing crystallinity and crystallization rate. The crystal structures were different: only the α-crystal phase was observed in nanocomposites rather than both α- and β-crystals, which appeared in pristine PEF. However, the thermal stability of the nanocomposites weakened as those containing GO showed a T_d_ of 370 °C. Terzopoulou et al. [128] prepared poly(propylene 2,5-furan dicarboxylate)/graphene nanocomposites and investigated the thermal decomposition kinetics and mechanisms. The thermal analysis showed that the graphene had no obvious influence on degradation, but resulted in a tiny increase in activation energy while the degradation mechanism was not affected by the presence of graphene. Poly(trimethylene 2,5-furanoate) (PTF) was also prepared as nanocomposites with few-layer graphene (FLG) via in situ polymerization [129]. Compared to the pristine PTF, the nano-reinforced composites exhibited improved mechanical properties and thermal stability. The crystallization behavior was not affected by the FLG. Achilias et al. [130] synthesized PEF composites with SiO_2_ and TiO_2_. The effect of nano-fillers on the SSPC was investigated and the presence of additives resulted in higher transesterification rate constants and lower activation energies. To enhance the slow crystallization of PBF, Zhou et al. [131] synthesized PBF/TiO_2_ nanocomposites via in situ polymerization with rutile/anatase TiO_2_. The crystallization rate of the composites obtained was less than 1 min at a TiO_2_ content of 7‰. Additionally, the mechanical properties were proven as the elongation at break and impact strength increased by 118% and 200%.

The well-dispersed clay on the polyester matrix could reinforce the thermal, mechanical, and barrier properties, but showed a possible negative impact on molecular weight and the thermal stability of the composites [132,133,134]. Organo-modified montmorillonite (OMMT) clays were employed in the preparation of PEF nanocomposites via the solvent casting method and the crystallization of the composite was improved due to the nucleation effect of the clays [135]. Additionally, the thermal stability was significantly enhanced since the initial degradation temperature was increased by 20 °C. Aluminosilicate clays were utilized in the preparation of PPF composites to enhance the crystallization rate of the pristine PPF [136]. The samples presented good thermal stability (>300 °C) and the β-scission was found to be the dominating mechanism in the degradation process. To improve the low crystallization rate of PEF, PEF/nanocrystalline cellulose composites were prepared via solvent casting and the sonication procedures were utilized to well-disperse the cellulose nanocrystals. The thermal measurements revealed a slight improvement in the thermal degradation of nanocomposites [137]. There are many reports highlighting the significance of nanocellulose in the reinforcement materials since they have high crystallinity and strong hydrogen bonds that apply greater barrier properties [138,139,140]. Further investigations have shown that PEF/cellulose composites prepared via twin screw extrusion exhibited faster crystallization, as expected [141]. Composites based on poly(butylene 2,5-furanoate), poly(butylene 2,5-furanoate)-co-(butylene diglycolate), and bacterial cellulose were investigated by Matos et al. [142]. Results showed that the T_g_ was in the range from −25 °C to 46 °C and T_m_ was between 61 °C and 174 °C. The composites obtained exhibited highly reinforced mechanical properties such as 1240 MPa for tensile modulus, 14 MPa for tensile strength, and elongation at break up to 240%. In addition, the barrier property of composites was superior to that of PBF. Other nano-clays such as organically modified sepiolite-based and montmorillonite-based PEF composites were prepared via melt extrusion [143]. The thermal stability was improved by the clays used while the thermal and rheological properties were unaffected.

### 5.2. Blends of Furanoates

It is well-known that reactive blending, which is applied to promote the miscibility between furanoates and other polymers at high temperature, is an efficient way to obtain homogenous polyester blends [144]. Poulopoulou et al. [145] prepared a series of polyester blends based on PEF, PBF, and PPF and their terephthalate homologs via reactive blending. The blends of PEF/PPF exhibited dynamic homogeneity and miscibility, but PPF/PBF and PEF/PBF showed immiscibility. The temperature and time were proven to be the crucial factor to control the miscibility of furan-polyester blends. Additionally, the composition and the catalysts play an essential part in the blending process. They further investigated the five different PBF blends with PLA, PET, PPT, PC, and poly(butylene 2,6-naphthalenedicarboxylate). The samples exhibited immiscibility except for PET/PBF. Moreover, they suggested that reactive blending can improve the homogeneity of immiscible blends such as PBN/PBF blends [146]. Interestingly, when the backbone of the two polyesters differed by a single methylene unit, the blends would probably be dynamically homogeneous, while being heterogeneous if differing by two methylene units [147]. They introduced poly(ethylene terephthalate-co-ethylene furanoate) to the PEF/PET blends and the miscibility of the blends was improved. Furthermore, they assumed that any furan-polyester blends with PC were essentially immiscible [148,149]. Long et al. [150] prepared PBF via transesterification and then blended it with PLA to make a composite in which PBF was dispersed from the sphere to fibril phase. Results showed that the elongation at break of the blends was 17 times better than the neat PLA, while the mechanical modulus and strength stayed the same. In addition, the impact strength was also enhanced compared to that of the pristine PBF and PLA. They further investigated the retroreflection in PLA/PBF blends and found that the separated phase or semi-crystallization were the main structure in the blends, which was compared to the tapetum lucidum in the cat’s eye by the authors [151].

## 6. Conclusions

Making materials from renewable resources is what a sustainable economy requires. Standing out from the bio-based polymers, furan-polyesters are on the way from academia to industry. While one of the crucial issues is the price of raw materials, FDCA, as produced from the oxidation of 5-hydroxymethylfurfural and mainly catalyzed by noble metals from biomass (fructose and lignin), cannot meet the requirement for industrial preparation. Therefore, efficient, economic, and cheap catalysts for FDCA production are urgently needed. As reviewed above, polyester materials based on FDCA present encouraging properties as they satisfy the thermomechanical property, which lays the groundwork for a broad application range, excellent barrier property for packaging materials, and biodegradability for a cyclic economy. Nevertheless, furanic polyesters still suffer from discoloration due to the substandard purification of FDCA and decarboxylation or other side reactions. An investigation of colorless transparent furanic polyesters was reported in 2021 [152]. Another is the unexpected susceptibility of PEF to UV irradiation, which showed obvious degradation signs such as discoloration, deteriorating thermal properties, and chain cross-linking [153]. In summary, with further synthetic advances and structural manipulation for designated properties, FDCA-based polyester materials are inspiring in a variety of areas.

## Figures and Tables

**Table 1:** Thermal and mechanical properties of furan-polyesters with aliphatic diols via different synthetic methods.

Polyester	Synthetic Method	T_g_ /°C	T_m_ /°C	T_dmax_ /°C	Tensile Modulus (GPa)	Tensile Strength (MPa)	Mn (10^3^ g/mol)	Ref.
PEF	Transesterification	77–80	214	398	2.45	35	22.4–83	[10,33]
PEF	Direct esterification	80–89	210.4	407	2.1	66.7	105.3	[11,35]
PEF	ROP	80	215	-	-	-	-	[32]
PPF	Direct esterification	53–57.9	180	396	1.6	68.2	13.9–60.2	[11,41]
PBF	Direct esterification	30.5–40	171	392–428	0.7–1.1	5.5–32.9	8.0–23.2	[11,21]
PHF	Direct esterification	28.1	148.2	389	0.5	35.5	32.1	[11]
POF	Direct esterification	21.8	148.6	391	0.3	20.3	20.7	[11]
POF	Transesterification	−5	140	-	0.3	26.5	34.6	[42]
Poly(nonylene 2,5-furanoate) (PNF)	Transesterification	−30	69	-	0.2	21	40.0	[42]
Poly(decylene 2,5-furanoate) (PDeF)	Transesterification	−8	116	-	0.2	11	36.7	[42]
Poly(dodecylene 2,5-furanoate) (PDoF)	Transesterification	−22	111	-	0.2	11	39.4	[42]

**Table 2:** Thermal and mechanical properties of furan-polyesters with rigid diols via different synthetic methods.

Polyester	Synthetic Method	T_g_ /°C	T_m_ /°C	T_d5%_ /°C	T_dmax_ /°C	Tensile Modulus (MPa)	Tensile Strength (MPa)	Mn (10^3^ g/mol)	Ref.
PDASF	Solution polycondensation	180	-	-	450	-	-	13.8	[10]
PDAIF	Solution polycondensation	140	-	-	396	-	-	5.7	[10]
PIsF	Transesterification	157	-	-	421	-	-	-	[58]
PDIsFs	Transesterification	−1–21	64–111	405–413	439–444	14–559	0.7–20	11.5–25.4	[59]
PBIF	Transesterification	45–105	160	339–357	365–384	-	-	24.4–31.0	[60]
PISBF	Transesterification	55–151	-	370–376	405–417	1470	63	9.3–19.1	[61]
PCIsFs	Transesterification	75–103	220–257	363–375	402–409	-	-	-	[62]
PIsI	Transesterification	73	-	274	310/382	-	-	2.6	[63]
PBIS	Direct esterification	−28–−11	89–109			64–87	17–23	45.7–53.5	[64]
PXIIF	Transesterification/SSPC	94	250		411	-	-	30.3	[65]
PCF	Transesterification	71–87	219–291		377–403	1.69–1.82	44–52	8.3–26.2	[23,66]
PCdF	Transesterification	175	-		380	-	-	6.5	[66]

**Table 3:** Thermal and mechanical properties of furan co-polyesters based on PEF.

Polyester	Synthetic Method	T_g_ /°C	T_m_ /°C	T_d5%_ /°C	T_dmax_ /°C	Tensile Modulus (GPa)	Tensile Strength (MPa)	Mn (10^3^ g/mol)	Ref.
PECF	Transesterification	80–85	206–225	365–404	393–417	2.3–3.2	60–72	27.6–32.0	[76,77]
PPCF	Transesterification	59–73	193–232	378–382	412–421	1.9–2.1	79–88	35–41	[80]
PETF	Transesterification	90.9–91.1	-	368–369	400–403	3.1–3.3	97–98	(M_v_ = 51–56)	[82]
PEFC	Direct esterification	32.3–76.1	-	365–390	394–421	0.8–1.8	18–49	18.8–22.7	[78]
PEFEG	Direct esterification	78.5–84.9	177–209	345–360	-	-	11–27	34.0–46.8	[83]
PEICF	Direct esterification	90–119	-	-	-	-	-	16.4–19.6	[81]

## Data Availability

The data presented in this study are available on request from the corresponding author.
